# Antipyretic Medication for a Feverish Planet

**DOI:** 10.1007/s41748-020-00182-6

**Published:** 2020-11-02

**Authors:** Markus Stoffel, David B. Stephenson, Jim M. Haywood

**Affiliations:** 1 Climate Change Impacts and Risks in the Anthropocene (C-CIA), Institute for Environmental Sciences, University of Geneva, 66 Boulevard Carl-Vogt, 1205 Geneva, Switzerland; 2 Dendrolab.ch, Department of Earth Sciences, University of Geneva, 13 rue des Maraîchers, 1205 Geneva, Switzerland; 3 Department F.-A. Forel for Environmental and Aquatic Sciences, University of Geneva, 66 Boulevard Carl-Vogt, 1205 Geneva, Switzerland; 4 College of Engineering, Mathematics and Physical Sciences, University of Exeter, Exeter, EX4 4QF UK; 5 Earth System and Mitigation Science, Met Office Hadley Centre, Exeter, EX1 3PB UK

As the coronavirus pandemic continues to unfold at a staggering pace, CO_2_ emissions are in for a sharp, if temporary, decline estimated at 7% of the 2019 annual emissions (Le Quéré et al. 2020; Carbon Brief 2020; Forster et al. 2020). Even if this reduction is substantial, it will not suffice to reach the 1.5 °C global temperature target of the 2015 Paris Conference of Parties Agreement (COP, Brown et al. 2019), as a reduction by 7.6% would be needed every year from today to reach net-zero emissions by 2050 (Sachs et al. 2016). Therefore, once the pandemic and ensuing economic lethargy are over, societies will need to make a crucial choice on how to reach the climate goals defined at the COP. Global emissions could resume if nations decided to lean heavily on fossil energy sources to rebuild their economies (Henry et al. 2020; Ou et al. 2020). Under different leadership, strong governmental support for clean energy could tilt major economies towards a greener, more climate-friendly direction (Barbier 2020; Carbon Brief 2020; Rosenbloom and Markard 2020; Andrijevic et al. 2020).

Back in 1992, the Intergovernmental Panel on Climate Change (IPCC) forecast carbon dioxide (CO_2_) concentrations under their ‘IS92a best guess’ scenario (Nakicenovic et al. 2003). These predictions have proved remarkably accurate; an analysis of the mean CO_2_ concentrations over the past thirty years from the two models available at that time (for details see IPCC 2020) indicates that they are never in error by more than 1.5 ppmv when compared to CO_2_ observations (NOAA 2020). CO_2_ concentrations are currently increasing at a rate of around 0.5% per annum; if this continues (as they have for the last 50 years; Showstack 2013), atmospheric concentrations will rise from around 411 ppmv at current levels (their highest for the last 3 million years) to 611 ppmv by 2100 (i.e. 411 ppmv × 0.5% annual increase × 80 years). The IS92a scenario, that has proved so accurate over the last thirty years, suggests an even more pessimistic 713 ppmv (Houghton et al. 1995; IPCC 2020). Given the remarkable validation and future projections of CO_2_ concentrations, humanity cannot say that they have not been warned of the impact that their activities are having. The scientific consensus is that, given current mitigation efforts, the Paris Agreement target of limiting Global Mean Surface Temperature (GMST) warming to 1.5 °C (or even 2 °C; Masson-Delmotte et al. 2018) above pre-industrial values will be missed. Even if global warming continues to increase at the current rate of around 0.2 °C per decade, which is below the climate projection levels, the 1.5 °C threshold will be exceeded by 2040–50 (Masson-Delmotte et al. 2018). The above facts unfortunately lead to the conclusion that some governments—rather than reducing emissions drastically—may soon start to consider implementing the unpalatable option of solar radiation management geoengineering (Parson 2017; Schubert 2019). Although it may be a foul-tasting medicine, it is considered to provide considerable relief from the ever-increasing catalogue of damaging extreme events (Jones et al. 2018; Irvine et al. 2019; Irvine and Keith 2020).

Clear evidence exists that human-induced GMST increases have already caused an increase in the frequency and intensity of heavy precipitation events at many locations across the globe, as well as a substantial increase of drought and flood risks in many arid and semi-arid regions (Myhre et al. 2019; Atif et al 2020; Tabari 2020; Yiwei et al. 2020). Assessments of projected future climate changes show that any further warming will increase the probability for unprecedented extreme weather and climate events (Masson-Delmotte et al. 2018; Myhre et al. 2019). While limiting global warming to 1.5 °C may seem only marginally different to limiting it to 2 °C, there are substantially larger probabilities of extreme events occurring under 2 °C scenarios (Kharin et al. 2018). Indeed, limiting warming to 1.5 °C above pre-industrial levels would (a) result in around half a billion fewer people being frequently exposed to extreme heatwaves, (b) reduce the risk of a further increase in frequency and intensity of heavy precipitation events, and (c) substantially reduce the probability of extreme drought and water scarcity (Masson-Delmotte et al. 2018). The number of category 4 and 5 tropical cyclones is also expected to increase in a warmer climate (Jones et al. 2017, 2018; Knutson et al. 2020). With global warming of 2 °C, risks across the energy, food, and water sectors will increase compared to the 1.5 °C target, overlapping spatially and temporally, and thereby exacerbate climate-induced hazards, exposures, and vulnerabilities. As a result, substantially larger proportions of people would become exposed and susceptible to poverty with further warming, especially in Africa and Asia (Masson-Delmotte et al. 2018). In addition, the IPCC also notes that rising GHG emissions will raise global sea level by more than a metre by 2100. Even under more favourable scenarios, cities, such as Los Angeles and Miami, might face a “100-year” coastal flood every year by 2050 (Masson-Delmotte et al. 2018). Should humanity fail to prevent the loss of major Antarctic and Greenland ice masses, future generations would see far worse. Furthermore, a majority of current climate models seem to underestimate the “extremeness” of impacts, namely in the agricultural sector, terrestrial ecosystems, or heat-related human mortality (Schewe et al. 2019). Given that without draconian mitigation strategies, in the authors’ opinions, both the 1.5 °C and 2 °C targets defined in the Paris Agreement are likely be missed, society will be exposed to increasing losses and disasters from more frequent and intense catastrophic weather extreme events, for example, events similar or even more powerful than the 2019 Hurricane Dorian and Typhoon Hagibis (Tay et al. 2020).

If greenhouse gas emissions are not reduced fast enough to avoid overshooting the Paris targets, then it seems not unlikely that future governments may well consider deploying “geoengineering” (also referred to as “climate intervention or “climate repair”); with the aim to decelerate the rate of global warming and to buy more time for mitigation and adaptation, thereby reducing risk from climate-related losses. One of the most promising approaches is Solar Radiation Management (SRM), which aims to reduce the amount of solar radiation reaching the Earth’s surface (The Royal Society 2009; Lawrence et al. 2018). Owing to considerations of effectiveness, cost, technical feasibility and timeliness (e.g. The Royal Society 2009), the two most widely discussed forms of SRM are the deliberate injection of aerosol particles or their precursors into the stratosphere (i.e. stratospheric aerosol injection, e.g. Robock et al. 2009; Kravitz et al. 2011) and the deliberate injection of aerosol particles into low-lying stratocumulus clouds, with the aim to increase their reflectivity (so-called marine cloud brightening, e.g. Jones et al. 2011; Latham et al. 2012; Stjern et al. 2018). Both processes are known to cool the planet and occur naturally via volcanic emissions of sulphate-aerosol-forming sulphur dioxide (SO_2_) that are either explosive (emissions into the stratosphere, e.g. Soden et al. 2002) or passive (emissions into the lower troposphere, e.g. Malavelle et al. 2017).

For the last decade, models used in assessing global warming have also been making increasingly sophisticated assessments of the likely impacts of SRM. Early experiments involved relatively simple idealized reductions in the solar output (i.e. simply turned down the sun) to offset the entire future global warming from increases in greenhouse gases (Kravitz et al. 2011, 2013), with assessments of reductions of climate extremes in multi-model ensembles (Irvine et al. 2019). While useful, these multi-model assessments are ultimately limited by their idealized treatment of SRM.

Only the most foolhardy would suggest utilizing offsetting an ever-increasing warming via injection of an ever-increasing veil of stratospheric sulphate aerosol. This approach is fallacious for two well-established reasons. First, sophisticated stratospheric aerosol modelling has revealed less and less cooling per unit of SO_2_ injection, therefore diminishing returns that underline the need for comprehensive decarbonization during any stratospheric aerosol injection deployment period (Niemeier and Timmreck 2015). Second, such a strategy will neglect the rapid return to the non-geoengineered climate should SRM be halted for any reason (Jones et al. 2013). Compressing the climate change that the Earth would experience under global warming into less than a decade would likely devastate many ecosystems (Trisos et al. 2018).

In addition to these two major roadblocks for offsetting a large amount of global warming by SRM, detailed modelling studies have elucidated how *NOT* to deploy geoengineering. It has been shown that any stratospheric aerosol injection strategy that targets one hemisphere in isolation will lead to changes in the cross-equatorial energy and moisture flows that could have potentially devastating consequences on tropical rainfall patterns and associated droughts, floods and hurricane frequency and intensity (Haywood et al. 2013; Jones et al. 2017). Efficient marine cloud brightening relies on increasing the cloud albedo through a combination of increasing their reflectivity (the first indirect effect) and increasing cloud fraction (second indirect effect) (Stjern et al. 2018). However, new observational evidence suggests that the second indirect effect would be strongly buffered in the climate system and that it would do little to contribute to increased reflection (Malavelle et al. 2017; Toll et al. 2019). Model results suggest that any marine cloud brightening strategy that targets a particular cloud deck can not only alter the meridional cross-equatorial energy and moisture flows but can induce changes in mean-zonal Walker-circulation flows as well, with potential devastating consequences for continental-scale rainfall (Jones et al. 2009). Based on the above, one has to conclude that deliberate marine cloud brightening has many drawbacks compared to deliberate stratospheric aerosol injection. Marine cloud brightening may be ineffective at reducing global mean temperatures, and even if it should be effective, it will tend to preferentially cool ocean areas rather than land areas, and any geographically inhomogeneous forcing may force undesirable dynamical changes in circulation and precipitation patterns (e.g. Jones et al. 2009, 2011).

Learning from past climate model simulations of how not to perform SRM, approaches have matured from idealized experiments to include assessments where the global warming from the various Representative Concentration Pathway (RCP) scenarios are limited to the specific climate targets of the Paris Agreement via stratospheric aerosol injections (Tilmes et al. 2016; Jones et al. 2018; Irvine et al. 2019; Irvine and Keith 2020). These single-model studies suggest that under virtually all Representative Concentration Pathway (RCP) scenarios, risks identified in key weather and climate extremes (e.g., water stress, heatwaves, or the number and intensity of large Atlantic hurricanes) are ameliorated significantly.

**Fig. 1:**
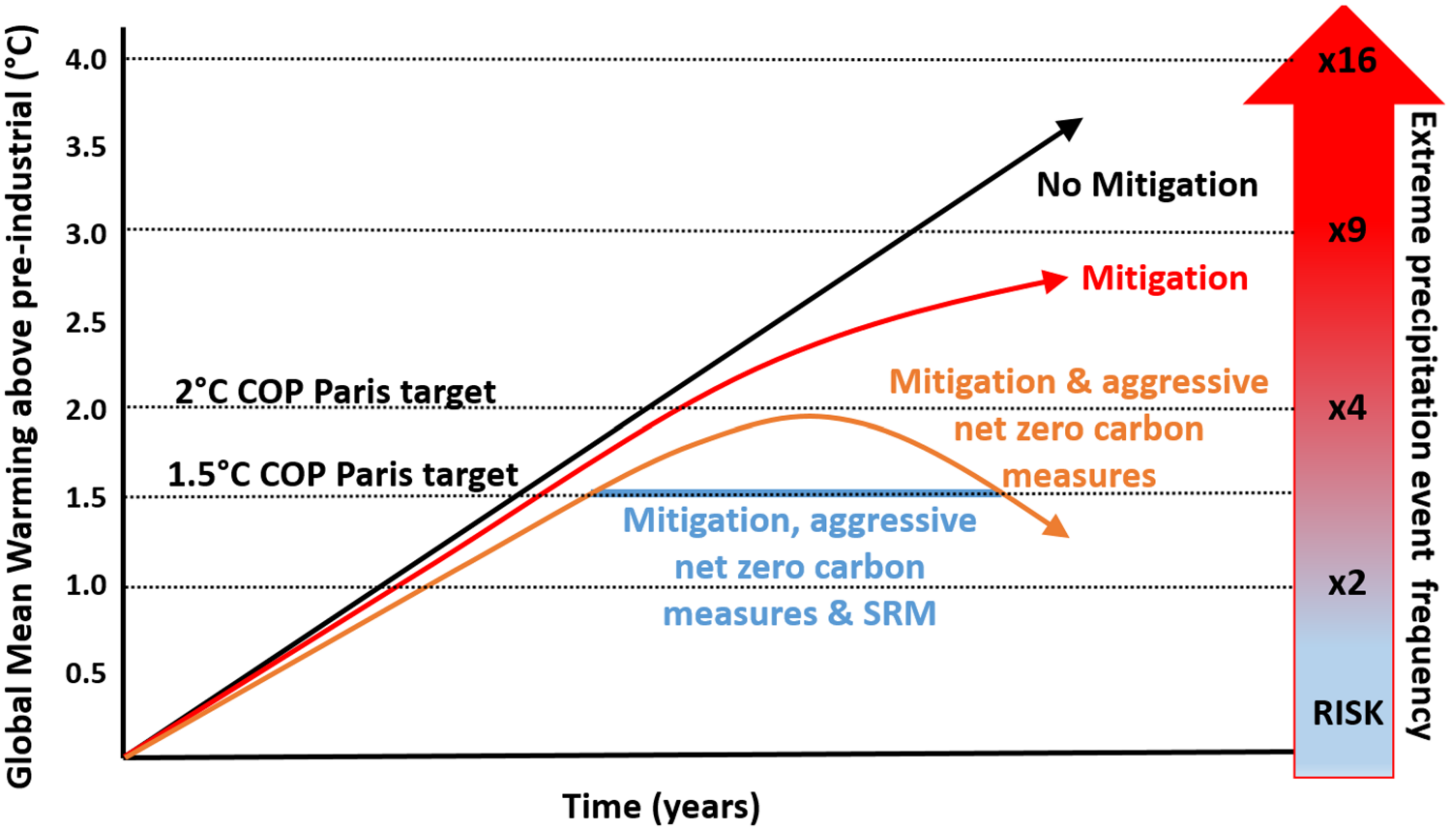
Global warming expected for no mitigation, current mitigation scenarios, mitigation including aggressive measures to achieve next zero carbon emissions and a mitigation, and the use of SRM to ‘peak shave’ global mean temperatures to maintain global mean temperatures at 1.5 °C as per the Paris COP target ( adapted from Jones et al. 2018). The analysis of risk in this case is based on the frequency of extreme precipitation per degree of mean global warming (Myhre et al. 2019)

Net zero carbon emissions clearly have to remain the goal to be achieved by humanity, as it remains the only way to mitigate climate change safely and sustainably in the long run and to reduce the plethora of risks which scale non-linearly with each degree of mean global warming (Fig. 1). However, given that the current mitigation activities are wholly incompatible with both of the Paris Agreement targets, governments will sooner or later have to acknowledge the need for back-up plans. In preparation for such an eventuality, it is entirely appropriate to develop model-based research into the physical science of SRM, to fill remaining gaps in scientific knowledge. The first step would be to accept failure at reigning in CO_2_ emissions despite decades of increasingly alarming evidence of global warming and its associated impacts. New Geo-Engineering Model Inter-comparison Project (GeoMIP) simulations with more sophisticated stratospheric sulphur cycle modelling coupled to more policy-relevant deployment scenarios are already underway (Jones et al. 2020; Kravitz et al. 2020). These model simulations are not only able to assess the amount of SO_2_ needed to achieve meaningful reductions in global mean surface temperatures, but also to assess the detailed regional and temporal response of the climate system at unprecedented detail. Many other aspects would need to be addressed from a scientific standpoint to increase confidence about possible and hitherto ignored side effects of SRM. These should include the search for potential alternatives to currently proposed marine cloud brightening and stratospheric aerosol injection strategies, state-of-the-art multi-model assessments of stratospheric aerosol injection scenarios in different seasons, different latitudes and altitudes, and at different emission rates. In addition, and despite obvious differences, comparisons of model outputs with observations from volcanic eruptions as natural analogues (Robock et al. 2010, 2013; Stoffel et al. 2015) would be a vital component, namely regarding the injection and evolution of SO_2_ into the stratosphere by explosive eruptions as an analogue for stratospheric aerosol injection or the natural release of SO_2_ during volcanic degassing events as an analogue for marine cloud brightening. Such natural events provide the necessary observational data for validating the fidelity of our models.

Even if models should reach sufficient quality, one would still need to determine the monetary costs of long-term efforts associated with the different SRM methods. From a purely socio-economic perspective, the development, deployment and operational costs obviously need better quantification, but the socio-economic costs that could be *avoided* by reducing the risk of damaging extreme floods, droughts, heatwaves or tropical storms also need evaluating. While an assessment of physical damage of such disasters can be assessed to reasonable accuracy with data from insurance industry, any assessment of the actions of humanity in response to an increase in extremes will be far less tangible and more difficult to assess. In the case that no political action is taken to limit global mean surface temperature warming to the targets defined in the Paris Agreement, the temperature and water availability of the Mediterranean may resemble that of the Sahara Desert by the end of the twenty-first century (Jones et al. 2018). Furthermore, under the RCP8.5/SSP5-8.5 scenario and using the same metric, much of Africa, Australia, Amazonia and South East Asia will experience climates that are outside the envelope of what currently exists on Earth, they have simply not been experienced in human history (Jones et al. 2018; Almazroui et al. 2020a, b). Under such circumstances, mass migration would seem inevitable with subsequent socio-economic impacts that are difficult to predict.

If there is even the slightest chance that governments will attempt to deploy SRM in future decades to limit global warming and its consequences, then there is a pressing need now to do more research in this area to develop a deeper understanding. In other words, if antipyretic medication might need to be administered, then there needs to be more detailed knowledge of its benefits and contraindications. In parallel, and as national lockdowns and confinements will come to an end after the COVID-19 pandemics, governments should take immediate measures and a modest fraction of the current global stimulus funds (Andrijevic et al. 2020) to speed up clean energy transitions, boost energy resilience and to put the world on track to achieve the Paris Agreement goals, so that humanity would come out of this crisis in a much better position than they were before.
